# Spatiotemporal Patterns of Sleeping Site Use of Guinea Baboon Parties (*Papio papio*)

**DOI:** 10.1002/ece3.71610

**Published:** 2025-07-27

**Authors:** Lisa Ohrndorf, Roger Mundry, Jörg Beckmann, Julia Fischer, Dietmar Zinner

**Affiliations:** ^1^ Department for Primate Cognition Georg‐August‐Universität Göttingen, Johann‐Friedrich‐Blumenbach Institute Göttingen Germany; ^2^ Cognitive Ethology Laboratory German Primate Center – Leibniz Institute for Primate Research Göttingen Germany; ^3^ Nuremberg Zoo Nürnberg Germany

**Keywords:** competition, habitat use, parasite load, predation risk, resource availability

## Abstract

Sleeping site selection in animals is thought to be influenced by multiple factors, including resource availability and competition, predation risk, thermoregulation and the risk of parasitic infection. While previous research has highlighted the importance of these factors, little is known about how they affect spatiotemporal patterns of sleeping site use among multiple groups of non‐territorial species with overlapping home ranges. Here, we investigated the sleeping site use of Guinea baboons ( *Papio papio* ) in Simenti, Senegal. We used locational data for 4 years of several baboon parties sharing the same range. We assessed the distribution of sleeping sites within the local habitat mosaic and patterns of sleeping site use of parties across time. We further investigated the proximity of sleeping sites of parties to those of co‐occurring parties and the impact of food availability and predator presence at the landscape level on the distance between parties' sleeping sites on the same night. In Simenti, Guinea baboons use trees exclusively as sleeping sites, and we found that the vast majority of sleeping sites were in the gallery forest along the perennial Gambia River, likely due to the availability of tall trees. Patterns of sleeping site use of individual parties in general showed no evidence of predator or parasite avoidance. Between parties, Guinea baboons slept in close spatial proximity (< 50 m) to at least one other party, regardless of food availability and predator presence. Thus, Guinea baboons in Simenti likely use the abundantly available sleeping sites opportunistically rather than being driven by intergroup competition or strategies for predator avoidance or reduction of the risk of parasite infection.

## Graphical abstract

Sleeping site selecti in primates is shaped by ecological and social factors, yet few studies have examined this in non‐territorial species with overlapping home ranges. Using 4 years of locational data, we show that Guinea baboon parties in Simenti, Senegal, consistently sleep in the gallery forest along the Gambia River and in close proximity to other parties, regardless of food availability or predator presence. Our findings suggest that sleeping sites are not a limited resource in this population and that sleeping site use is largely driven by opportunism.

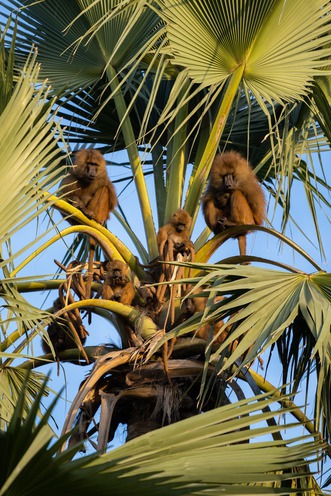

## 1 Introduction

Choosing suitable and safe sleeping sites is considered critical to the fitness of animals (Lewis 1995; Beauchamp 1999; Lima et al. 2005; Smeltzer et al. 2022). Sleeping site choice is often seen as an optimisation process with which individuals or groups balance the effects of social and ecological factors, including resource availability and competition (Chapman 1989), predation risk (Anderson 1984), exposure to parasites (Hausfater and Meade 1982; Anderson 1998; Zeus et al. 2017), territory and range defence (Heymann 1995), thermoregulation (Anderson 1998; Beauchamp 1999; Samson and Hunt 2012) and social cohesion (Papageorgiou et al. 2019; Bordes et al. 2022; Loftus et al. 2024). Depending on the species and the ecological conditions in their habitats, the importance of these factors varies, and animals are expected to select sleeping sites with specific beneficial attributes, for instance, in colder habitats, thermoregulation might be more important than distance to water or food resources.

While these factors shape sleeping site selection across taxa, they are particularly well‐studied in primates. Most primate species are diurnal and mostly inactive at night, making them vulnerable to nocturnal predation (Cowlishaw 1994). Many rely on specific landscape features like tall trees, cliffs, or rocky outcrops as safe sleeping sites (W. J. Hamilton 1982; Anderson 1998); they often exhibit strong site fidelity within their home ranges and reuse a consistent set of sleeping sites across nights (Jolly and Pride 1999; Bidner et al. 2018). Typically, in group‐living primates, sleeping sites accommodate multiple individuals or groups of individuals (Schreier and Swedell 2008; Markham et al. 2016), providing opportunities to study how social and ecological pressures affect patterns of sleeping site selection.

For diurnal, group‐living primates, a key factor influencing sleeping site selection may be predation risk, making safe sleeping sites essential for protection from predators. Various primate species prefer sleeping sites with specific attributes, such as trees that are tall, have large diameters at breast height (DBH) or difficult‐to‐climb trunks that likely offer better protection from nocturnal predation (e.g., titi monkeys *Callicebus coimbrai* ; Souza‐Alves et al. 2011; black‐fronted titi monkeys *C. nigrifrons* ; Caselli et al. 2017; Bornean agile gibbons *Hylobates albibarbis* ; Cheyne et al. 2012).

A second important factor for these species may be the distance to food resources that allows individuals to spend more time feeding by minimising travel distances and associated costs between sleeping sites and feeding patches (Sigg and Stolba 1981; Schreier and Swedell 2008; Henriquez et al. 2021; Chapman 1989; Albert et al. 2011; Teichroeb et al. 2012).

The availability of sleeping sites with specific attributes may vary considerably between habitats. Species inhabiting forested areas that sleep openly on the branches of tall trees might find an abundance of suitable sleeping sites to choose from in their habitat. In contrast, other species or populations that live in more open habitats may have access to only a few sites within their home ranges (e.g., hamadryas baboons *P. hamadryas* ; Kummer 1968; Sigg and Stolba 1981). For such species, suitable or high‐quality sleeping sites are often considered a limited resource, similar to species that sleep in tree holes. A scarcity of suitable sleeping sites may lead to competition for such sites among groups or subgroups that share a home range (Altmann 1974; Cheyne et al. 2012; Markham et al. 2016).

When multiple suitable alternatives are available, animals may employ a strategy of switching between sleeping sites to respond to ecological and social pressures. For example, switching regularly between multiple sleeping sites may facilitate access to patchily distributed or seasonally fluctuating food resources, especially when foraging areas shift over time. Switching sleeping sites may also reduce the predictability of an individual's or group's nocturnal location, thus decreasing the likelihood of being detected by predators (W. J. Hamilton 1982; Mitchell and Lima 2002; Markham et al. 2016; Caselli et al. 2017).

Another often mentioned hypothesis is that animals may switch between sleeping sites to minimise exposure to faecal matter build‐up, which could otherwise increase the risk of parasitic infection (Hausfater and Meade 1982; von Hippel 1998).

Both the predictability of nocturnal locations by predators and the accumulation of faecal matter suggest that the quality of sleeping sites should decrease when used continuously (W. J. Hamilton 1982; Markham et al. 2016). Patterns of sleeping site use in line with parasite avoidance have been found in yellow baboons ( *P. cynocephalus* ) in Amboseli, Kenya (Hausfater and Meade 1982; Markham et al. 2016). However, the intervals of vacancies for sleeping sites of individual groups were shorter than the predicted optimum to avoid parasite contact, and the occupancy was almost constant when looking at sleeping sites shared among groups. This finding suggests that other factors, such as competition for high‐quality sites, proximity to feeding sites, or social factors, may play an essential role in sleeping site selection, particularly in non‐territorial species with overlapping home ranges.

In non‐territorial species with overlapping home ranges, sleeping sites are likely to be used by several groups or subgroups simultaneously or successively. This shared use may lead to competition over specific sites themselves or over nearby food resources. It may influence exposure to predators or parasites, especially when predictability or faecal accumulation increases with repeated use. At the same time, shared use of sleeping sites may provide benefits, such as reducing individual predation risk through dilution effects (W. D. Hamilton 1971; van Schaik 1983; Krause and Ruxton 2002) or enabling social interactions across groups. Thus, examining the simultaneous use of sleeping sites by multiple groups, particularly under temporally variable ecological conditions, may offer valuable insights into the social and spatial strategies animals use to balance these competing pressures.

In this study, we investigated the spatiotemporal patterns of sleeping site use by Guinea baboon parties in the Niokolo‐Koba National Park, Senegal. Guinea baboons ( *Papio papio* ) are group‐living, non‐territorial primates that are highly spatially tolerant, both within and between gangs (Ohrndorf et al. 2025). They form large multi‐male, multi‐female aggregations of 20 to more than 300 individuals that often gather at shared sleeping sites (Dunbar and Nathan 1972; Sharman 1982; Galat‐Luong et al. 2006; Patzelt et al. 2011).

**FIGURE 1:**
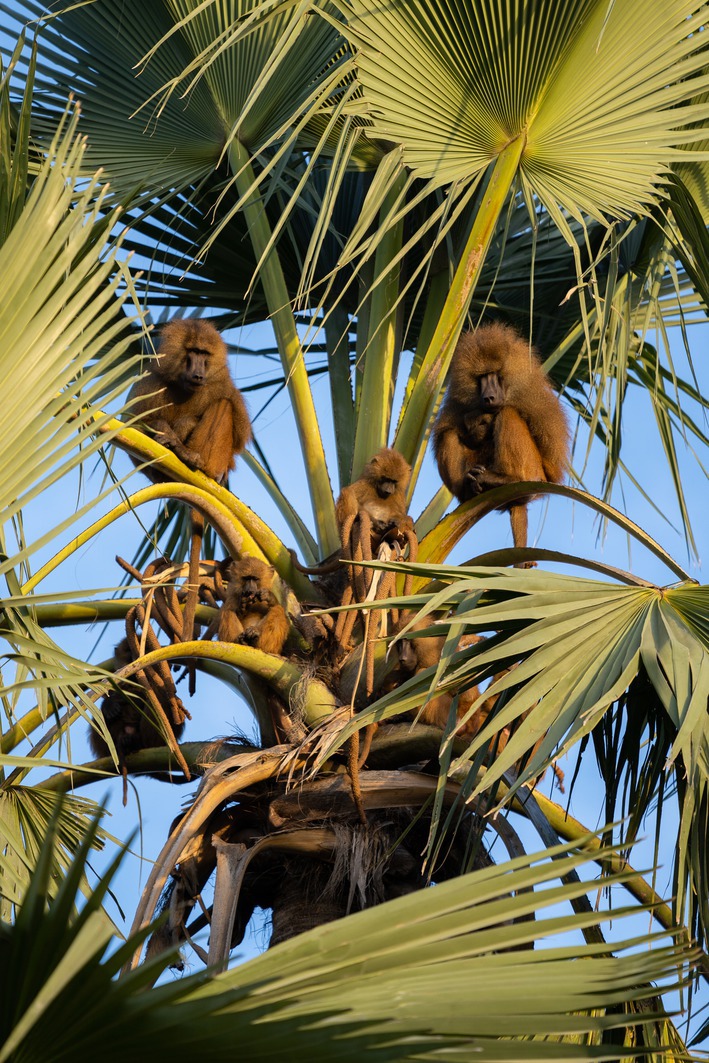
Guinea baboons using a Borassus palm (*Borassus akeassii*) as a sleeping tree in a tree/shrub savannah habitat. Picture taken by Tessa Ohrndorf.

Guinea baboon societies exhibit a nested multi‐level social organisation with fission‐fusion dynamics. At the core of this social organisation is the one‐male unit, consisting of one primary male, one to several females, their dependent offspring, and sometimes secondary males (Goffe et al. 2016). Several one‐male units form a party, which, together with other parties, form gangs (Fischer et al. 2017). In the Niokolo‐Koba National Park, the home ranges of parties vary between 20 and 50 km^2^ (Sharman 1982; Zinner et al. 2021) and almost entirely overlap with those of other parties belonging to the same gang, but also between gangs (Ohrndorf et al. 2025; Zinner et al. 2021). Most sleeping sites of Guinea baboons near our field station consist of one or several trees of *Ceiba pentandra* , *Celtis integrifolia* and *Borassus akeassii* (Figure 1), abundant in habitats bordering perennial water bodies. Like other baboon species, Guinea baboons are eclectic omnivores that primarily feed on fruit but also other plant parts, invertebrates and occasionally small birds or mammals (Sharman 1982; Zinner et al. 2021; O'Hearn et al. 2024). The most important predators of Guinea baboons in Simenti are African lions ( *Panthera leo* ) and leopards ( *P. pardus* ), but they also react very strongly to the presence of spotted hyenas ( *Crocuta crocuta* ) (personal observation). Both food availability and predator presence likely vary spatially and temporally across the landscape and within different habitat types used by baboons.

Using locational data from 4 years, we conducted an exploratory analysis of the spatiotemporal patterns of sleeping site use of Guinea baboon parties in Simenti, Senegal. We describe patterns of sleeping site use relative to habitat type in which sleeping sites were located. We further investigated patterns of sleeping site use of individual parties to assess whether we could find any pattern indicative of predator or parasite avoidance. Additionally, focusing on data collected in 2022, we examined the spatial relationships between sleeping sites of different parties that share the same range, testing whether distances between parties varied in response to variation in food availability and predator presence. Specifically, we predicted that parties would sleep closer to other parties when predator presence was high, as sleeping more closely to other parties likely offers advantages regarding predator detection and defence. Conversely, we predicted that parties would sleep further apart when food availability was low to mitigate feeding competition between parties when foraging in the evenings and mornings. With this, we aim to provide additional information on the drivers of sleeping site use in Guinea baboon parties.

## 2 Materials and Methods

### 2.1 Study Site

The study took place at the long‐term field site of the Centre de Recherche de Primatologie (CRP) in Simenti, Senegal (13°01′34′′ N, 13°17′41′′ W). The site is located in the Niokolo‐Koba National Park in southeast Senegal and belongs to the Sudanian and Sahelo‐Sudanian climatic zone (Arbonnier 2002). The vegetation in the study area around Simenti is classified as a mosaic of grasslands, wooded savannahs and gallery forests alongside rivers and other perennial water bodies (Arbonnier 2002; Burgess et al. 2004). Seasonality in this climatic zone is pronounced, with an annual precipitation of 950 mm, mainly limited to the rainy season. The rainy season typically lasts from June to October, with May and mid‐October constituting transitional periods with highly variable rainfall (Zinner et al. 2021). Several habituated Guinea baboon parties from different gangs have been followed extensively since 2007 for behavioural studies at our study site.

### 2.2 Data Collection

#### 2.2.1 GPS Data

To determine the location of sleeping sites of Guinea baboon parties within the study area, we deployed GPS collars (Tellus 2 Basic Light) with built‐in drop‐off mechanisms on 8 adult male Guinea baboons from 8 different parties (P). Each male represented their entire party's location (P5, P6I, P6W, P7, P9B, P13, P15, P17), with party membership determined through regular observations of social interactions and spatial proximity (Patzelt et al. 2014). Party membership remained stable for all collared individuals during the collaring interval. We programmed the collars to take locational fixes at 2‐h intervals during the day (06:00–18:00) and an additional 3 fixes during the night (21:00, 00:00, 03:00). For details on the collaring procedure, please see the Supporting Information.

**FIGURE 2:**
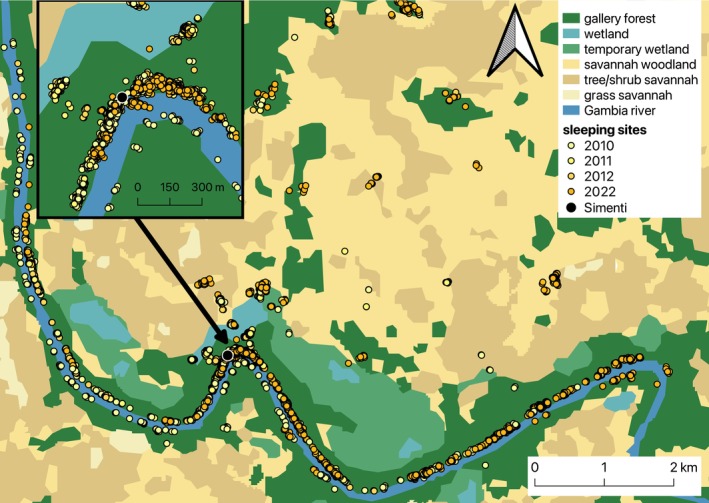
Locations of sleeping sites (spatial centre of GPS fixes taken at 21:00, 00:00 and 03:00 each night) in different habitat types within the study area for all parties surveyed from 2010 to 2012 and in 2022. The close‐up shows the extensive use of sleeping trees in the gallery forest along the Gambia River.

To identify the locations of the parties' sleeping sites, we subset the dataset to include only locational fixes taken at night. For each party and night, we calculated the pairwise distances between up to 3 successive locational fixes. If all successive points were within 100 m of the previous one, we estimated the sleeping site as the geographic centre of those points. We excluded locational points that were more than 100 m from the previous point and calculated the mean centre from the remaining point(s). Nights for which all points were more than 100 m apart were excluded from further analysis (Figure 2). The distance between the remaining locational fixes taken at 21:00, 00:00 and 03:00 for each party was 8.3 m on average (median, range 0–99.5 m), which may be caused by nocturnal movements of collared individuals or by GPS inaccuracies. We refer to ‘sleeping sites’ for a party as the nocturnal locations of the collared individual rather than as discrete, identifiable sites, as it was not possible to delineate such sites in the continuous gallery forest. This is because the study parties appeared to use nearly the entire extent of the forest along the Gambia River for sleeping, making it difficult to delineate clusters of trees or nocturnal locations as separate sleeping sites due to substantial spatial overlap (Figure 2).

Additional GPS data were available from a previous study for 11 individuals (4 females, 7 males) from 6 parties (P4, P5, P6, P8, P9, PU) in 2010, 7 individuals (2 females, 5 males) from 6 parties (P4, P5, P6, P8, P9, PU) in 2011 and 5 individuals (1 female, 4 males) from 5 parties (P4, P6, P8, P9, PU) in 2012 (Patzelt et al. 2014). The sampling intervals for these collaring periods were identical to those in 2022. In total, we collected the geographical coordinates of 8877 sleeping sites across all years and parties. In 2022, GPS collars lasted an average of 316 days, resulting in 2518 sleeping site locations across 8 study parties. One collar (P6I) failed after 162 days due to water damage.

#### 2.2.2 Food Availability

To evaluate food availability across our study parties' home ranges, we used the same phenological data as described in Ohrndorf et al. (2025). We conducted monthly phenological assessments of key feeding tree species from November 2021 to March 2023. We selected an average of 10 trees (range: 1–13) from each of the 31 most common feeding species identified by Zinner et al. (2021). These marked trees were monitored at the start of each month throughout the 17‐month study period. We recorded 8 phenological states: none, presence of young leaves, flower buds, flowers, young fruits, intermediate fruits, ripe fruits and mature leaves. Trees with ripe or intermediate fruits were classified as ‘providing food’, as were flowering trees of species known to be consumed by our study population. We calculated food availability at the species level as the proportion of trees providing food relative to the number of trees monitored for a given species. Then, we determined monthly food availability as the average of the proportions across all monitored tree species.

$$\text{Monthly food availability}=\frac{\sum_{i=1}^{N_{\text{species}}}\left(\frac{N_{\text{fruiting}}+N_{\text{flowering}}}{N_{\text{trees}}}\right)}{N_{\text{species}}}$$

#### 2.2.3 Predator Presence

We assessed predator presence across the study area using unbaited, motion‐triggered camera traps distributed across 37 km^2^ as described in Ohrndorf et al. (2025), covering most of our study parties' home ranges. We deployed the cameras on a grid of 1 × 1 km, with roughly one camera per km^2^. The imagery obtained from this camera‐trapping grid was annotated using the online platform *Agouti* (Casaer et al. 2019). We checked the AI‐assisted annotation manually (Brouillet 2024). We considered sightings of lions, leopards, hyenas, and one sighting of African wild dogs ( *Lycaon pictus* ) within the camera‐trapping grid as predator encounters.

In addition to the camera trap data, we collected ad libitum data on all signs of predators (tracks, scat, calls and sightings) during the study period. From both, camera trapping and ad libitum observations, we calculated the number of predator encounters in the study area in intervals of 14 days. As we could not be certain about the most relevant time window for assessing perceived predation pressure in Guinea baboons, we also calculated the number of predator encounters over shorter (2 and 7 days) and longer (30 days) intervals.

#### 2.2.4 Habitat Types

To understand in which habitat types sleeping sites of Guinea baboon parties predominantly occurred from 2010 to 2012 and in 2022, we used a supervised habitat classification based on multispectral Landsat 5 TM imagery from 28 November 2010 (Klapproth 2010) (Figure 1). This classification distinguished 6 physiognomic habitat types (gallery forest/forest, savannah woodland, tree/shrub savannah, grass savannah, temporary wetlands and wetlands) and focused on a 158 km^2^ area in the Simenti region, defined as the extent of all baboon locations recorded between 2010 and 2012, delimited using minimum convex polygons following Johnson (1980). We assessed the number of sleeping sites within each of the 6 habitat types and investigated whether the use of different habitat types for sleeping sites changed within and across years.

#### 2.2.5 Proximity Among Sleeping Sites of Parties

**FIGURE 3:**
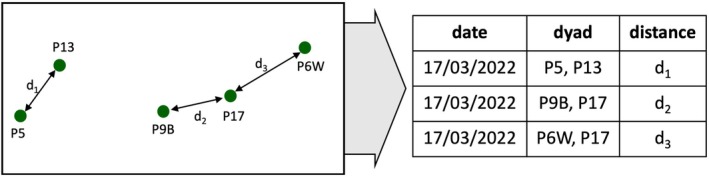
Example of the assessment of minimum distances between sleeping sites of parties. In this example, GPS data were available for 5 parties (P5, P6W, P9B, P13, P17). We identified the closest party dyads (P5 and P13, P9B and P17, P6W and P17) and the Euclidean distances between them (*d*_1_–d_3_).

To investigate patterns of proximity between the sleeping sites of our study parties in 2022, we assessed the minimum Euclidean distance between the sleeping sites of pairs of collared individuals, each representing their entire party's location, for each night when GPS data were available for both individuals. Therefore, we calculated all inter‐party distances for nights with available data and then selected the shortest dyadic distances such that each party was represented at least once per night it was observed simultaneously as another party (Figure 3).

We further used this dataset of minimum distances between sleeping sites of parties to assess which parties slept closest to each other most often. We chose to use minimum distances between parties instead of average distances because, in a limited space, sleeping sites that were further away from those of one party would likely be closer to the sleeping site of another, leading to averages not being able to capture the spatial relationships we were interested in. We focused our analysis on the proximity to the closest sleeping site of another party to avoid relying on arbitrary distance cut‐offs when assessing the number of parties in proximity, thus essentially dichotomising a continuous variable. This approach provides a more accurate and biologically meaningful representation of proximity, relevant for understanding how parties might adjust their sleeping site use in response to nocturnal predation risk or competition for food resources during the day.

In 2010–2012, several individuals per party were equipped with GPS collars, providing simultaneous locational data for multiple members of the same party. We used this data to estimate average minimum distances between sleeping sites of party members as a reference for within‐party distances.

### 2.3 Data Analysis

#### 2.3.1 Patterns of Sleeping Site Use of Individual Parties

**FIGURE 4:**
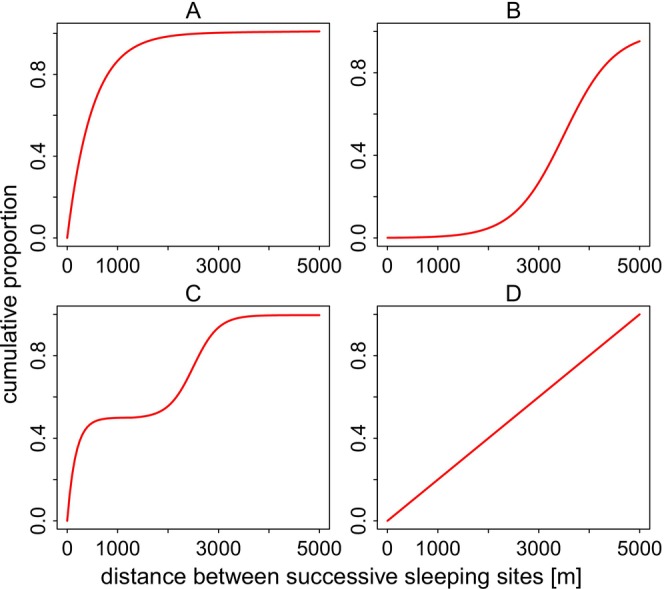
Cumulative proportion of distances between successively used sleeping sites under 4 hypothetical scenarios (A–D). The *x*‐axis represents the distance between successive sleeping sites. The *y*‐axis shows the cumulative proportion of data, indicating the percentage of the total data as distance increases.

Across all years of data collection (2010–2012, 2022), we visually inspected how individual parties used sleeping sites by plotting the distances between successive sleeping sites across time. We then randomised the order in which each party visited their sleeping sites 1000 times to see whether the observed distribution of distances between sleeping sites differed from the random expectation. We considered 4 hypothetical scenarios, each associated with different ecological or social drivers:

- *Local site fidelity driven by predictable resources or preference*: Guinea baboon parties might prefer to use sleeping sites close to those used the previous night, with only a few larger distances between successively used sites, indicating frequent use of a few select sites or overall mainly small‐scale replacement of successive sleeping sites (Figure 4A). This pattern may reflect stable local food availability near sleeping sites, low predation risk, or pronounced preference for specific sites.
- *Regular displacement for predator or parasite avoidance*: Parties consistently move moderate to long distances between successive sleeping sites and avoid reusing previously used sites (Figure 4B). This pattern could reflect a strategy to minimise the predictability of their nocturnal location to predators or reduce the risk of parasite infection associated with faecal matter build‐up at previously used sites.
- *Local fidelity with occasional large‐scale relocations*: Alternatively, we might observe a bimodal pattern, with many short distances on days when Guinea baboons reuse the same or nearby sites, combined with a considerable fraction of long distances observed when they relocate to areas far from previously used sites (Figure 4C). This mixed‐use pattern may reflect a flexible strategy that integrates the benefits of local site fidelity, such as access to predictable food resources, with the need to occasionally move away to reduce the risk of predator detection or contamination, or in response to disturbance by predators.
- *Random patterns of sleeping site use*: Guinea baboons might use sleeping sites randomly, visiting sites at all distances from previously used sites with equal likelihood (Figure 4D). This pattern would suggest that factors like local food availability, predator presence, or risk of parasitic infection have little to no influence on the spatiotemporal patterns of sleeping site use by individual parties.

#### 2.3.2 Effect of Food Availability and Predator Presence

To investigate whether the distance between sleeping sites of study parties varied with food availability and predator presence, we exclusively used data from 2022. There were no data available on food availability or predator presence for 2010–2012. We fitted multiple membership models using the function *brm* of the package *brms* version 2.20.4 (Bürkner 2017) in R version 4.3.1 (R Core Team 2023). We log‐transformed the minimum distance between parties per night to increase the probability of model convergence and included the log‐transformed distance as the response variable in our models. We included the monthly food availability score and the number of predator encounters within 14 days prior to the observation as predictors. To account for variation between individuals and dyads of individuals, we included the IDs of both individuals in a dyad (as a multi‐membership term) and dyad ID as random intercepts effect. We also included random slopes for food availability and predator presence within the 14‐day period to avoid overconfident model estimates (Barr et al. 2013; Schielzeth and Forstmeier 2009).

The multi‐membership approach estimates a single random intercepts effect for both individuals in a dyad. Thus, this random intercepts effect accounts for certain parties to be closer (or more distant) to other parties in general. We chose a multi‐membership model as the two individuals of a dyad could not be unambiguously assigned to two different random effects variables. The random intercepts effect of dyad, in turn, accounts for preferential associations between certain parties. Before fitting the model, we checked for sufficient variation (for covariates: at least 3 unique values per level of the grouping factor or at least 2 unique values, each with at least 2 observations per level of the grouping factor; for factors: at least 2 levels, each with at least 2 observations) of food availability and the number of predator encounters within each individual and dyad. As all predictors showed sufficient within‐group variation, we included random slopes for all fixed effects within both dyad and party. We also included parameters for the correlations among random intercepts and random slopes in the model. As we received a warning about divergent transitions during warm‐up with the default settings of *brm*, we set adapt_delta to 0.99.

To ensure that our model results were not biased by an inappropriate choice of the time window used to assess predator presence, we fitted 3 additional models, including the number of predator encounters per 2, 7 and 30 days instead of 14 days as predictors, but maintaining an otherwise identical structure.

## 3 Results

### 3.1 Sleeping Sites and Habitat Types

**FIGURE 5:**
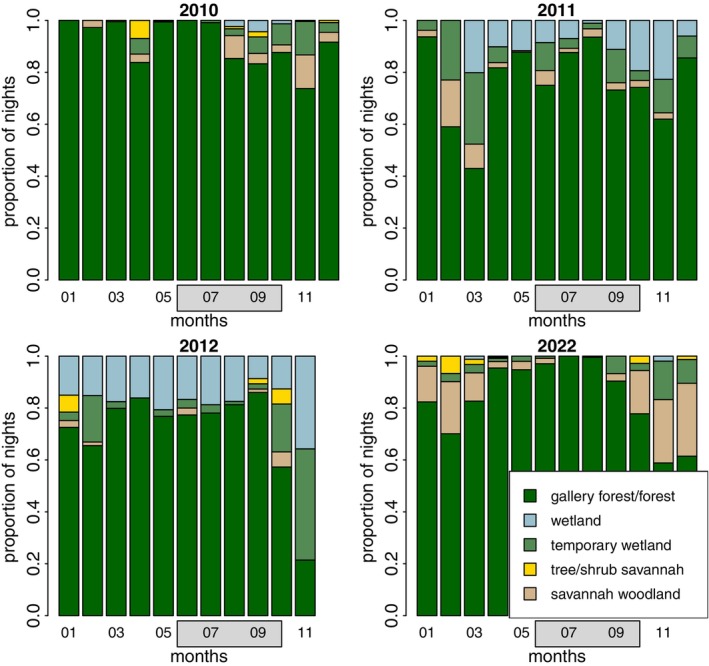
Proportion of nights spent in each habitat type per month across the years of observation. Grey boxes represent the months of the rainy season (June to mid‐October).

Out of 8877 recorded sleeping sites, 7413 were located in gallery forests and other forest types (84%), 473 in wetlands (5%), 444 in temporary wetlands (5%), 430 in savannah woodlands (5%) and 78 in tree/shrub savannahs (1%). One sleeping site fell outside of the geographic range of the habitat classification (Klapproth 2010) and could, therefore, not be assessed. Across all years, our study parties spent 84% of their nights in the gallery forest and other forest types, despite these habitat types covering only 15.5% and 14.8% of the geographic range of habitat classification, respectively (Klapproth 2010) (Figure 5). Our study parties spent 10% of their nights in trees near wetlands or temporary wetlands. When examining habitat use for sleeping site locations over the years, most sleeping sites were found in the gallery forests year‐round, particularly during the rainy season (June–October). During the dry seasons, there was more variability in habitat use, with parties spending more nights in other habitats such as wetlands, temporary wetlands, or savannah habitats. In 2022, our study parties spent substantially more nights in savannah woodlands during the dry season than during the rainy season (Figure 5).

### 3.2 Food Availability and Predator Presence

**FIGURE 6:**
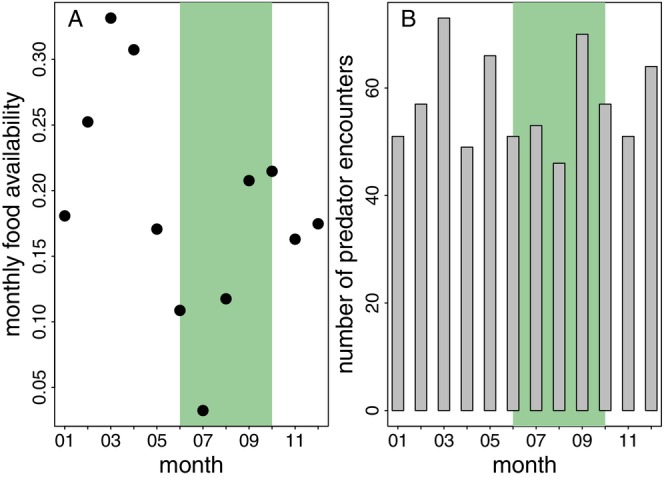
Monthly food availability scores for 2022 (A) and an overview of the number of predator encounters per month (B). The green area indicates the rainy season (June to mid‐October).

Food availability scores fluctuated between 0.03 and 0.33 from January to December 2022 (Figure 6A). The highest scores were observed in February, March and April 2022, while the lowest scores occurred in June, July and August, despite these months falling within the rainy season. In 2022, we recorded a total of 588 predator encounters (Figure 6B). This included 376 ad libitum records and 212 images of predators taken by camera traps. Spotted hyenas were the most frequently recorded predators (254 encounters), followed by lions (211) and leopards (118). Additionally, there was one sighting of a pack of 11 African wild dogs and 4 records that could not be reliably identified as either leopard or lion.

The average number of predator encounters varied depending on the time interval chosen to estimate predator presence. In 14‐day intervals, we recorded 25 predator encounters on average (median, range 17–42). The average number of predator encounters in 7‐day intervals was 13 (median, range 7–27). In 30‐day intervals, the average number of predator encounters was 53 (median, range 39–82) and in 2‐day intervals, it was 3 (median, range 2–13).

### 3.3 Patterns of Sleeping Site Use of Individual Parties

**FIGURE 7:**
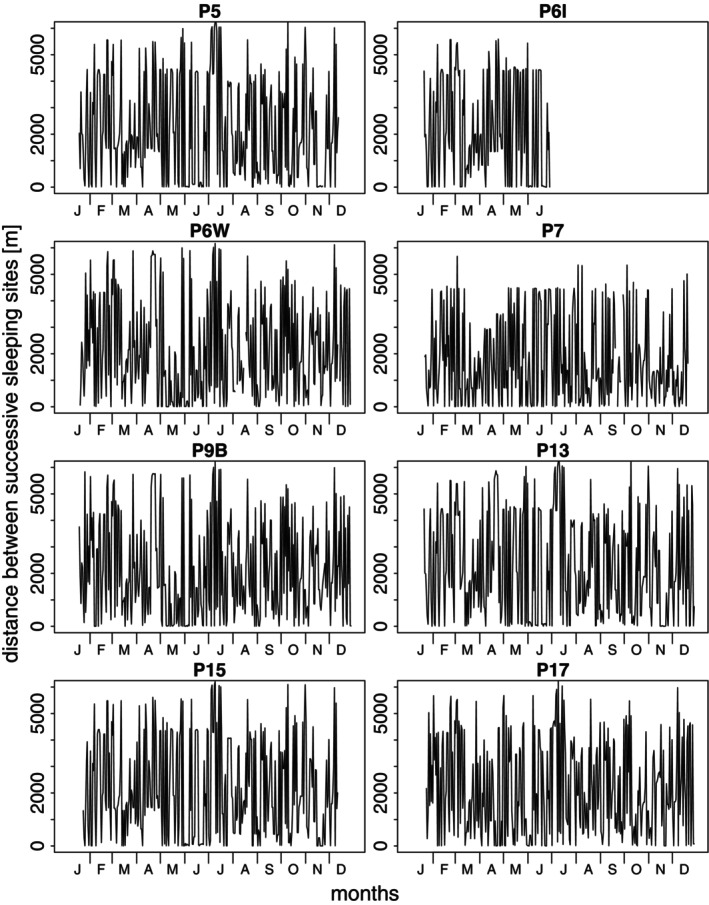
Distances between successive sleeping sites for all 2022 study parties across time. The collar for P6I failed in June 2022 due to water damage.

**FIGURE 8:**
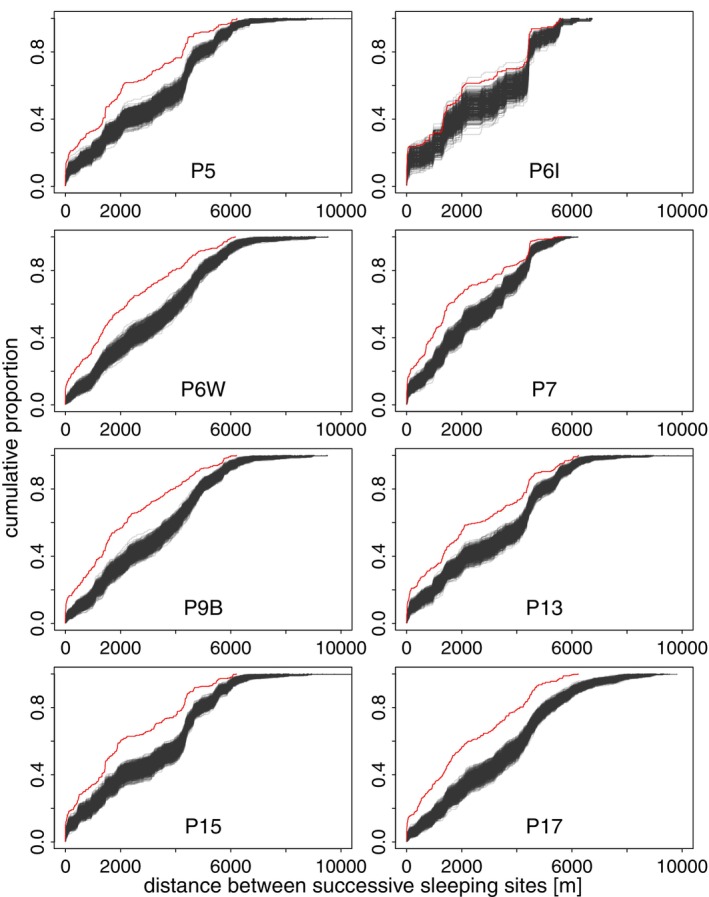
Patterns of sleeping site use of parties in 2022. Grey lines indicate the distance between successively used sleeping sites in 1000 randomised visit orders, and the red line represents the observed distances between successive sleeping sites.

Guinea baboons used sleeping sites that were, on average, 976.9 m from their previous sleeping site (median, range 0.2–7883.7 m). Visual inspection of the distances between successively used sleeping sites for each party indicated that sites were rarely used continuously for extended periods (Figure 7). Comparing observed patterns of sleeping site use with random permutations of visit orders showed a tendency for more shorter and intermediate distances between sleeping sites than would be expected by chance. However, the observed patterns were not markedly different from random expectations (Figure 8; Figures S1–S3).

### 3.4 Distance to Other Parties

**FIGURE 9:**
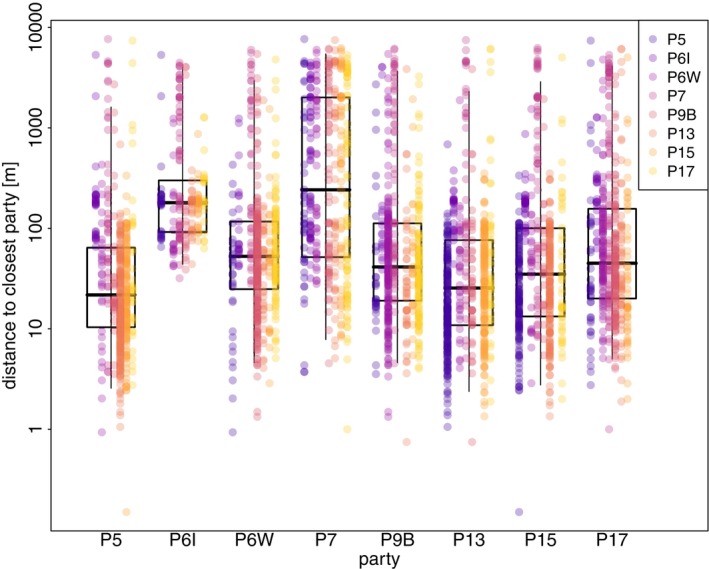
Minimum distances between sleeping sites of parties in 2022. Boxplots show the median (black line) and IQR with the lower (25%) and upper (75%) quartiles. Whiskers represent the 2.5th and 97.5th percentiles. The coloured points are overlaid to indicate, for each party, the identity and distance to the nearest party's sleeping site. The distance to the closest party is depicted on a log scale for visual clarity.

**FIGURE 10:**
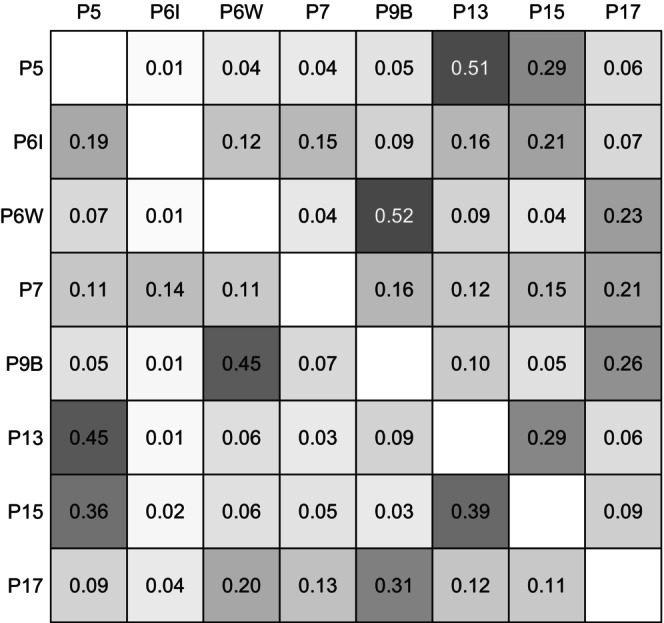
Asymmetric association matrix showing, for each party (rows), the proportion of nights that the other party (columns) slept closest to it. Cell shading reflects the proportion of nights that parties slept closest to each other (white = 0, black = 1), providing a visual guide alongside the printed values. Rows sum to 1. Note that associations are directional: If Party A's sleeping site was closest to Party B's, this does not imply that Party B's sleeping site was closest to Party A's.

The minimum distance between sleeping sites of Guinea baboon parties in 2022 was 47.9 m on average (median, 16.7–136.1 m IQR) (Figure 9). For comparison, the average minimum distance between individuals from the same parties in 2010–2012 was 11.8 m (median, 6.1–24.6 m IQR). Most parties consistently slept closest to one or two specific parties across nights. In contrast, P6I and P7 exhibited more variability in their nearest neighbours, with no clear preference for consistently sleeping close to any particular party (Figures 9 and 10).

**FIGURE 11:**
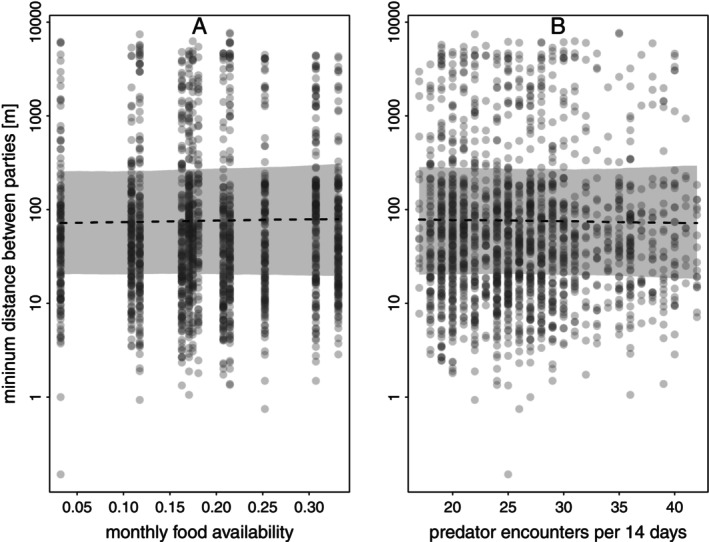
Model results for the effect of food availability (A) and the number of predator encounters per 14 days (B) on the minimum distance between sleeping sites of parties in 2022. For (A), predator presence was centred to a mean of zero, and for (B), food availability was centred to a mean of zero. The minimum distance between parties is depicted on a log scale for visual clarity. The posterior mean is depicted as a dashed line, and 95% credible intervals are shaded in grey.

We did not find evidence for an effect of food availability or predator presence on the minimum distance between sleeping sites of our study parties (Table 1; Figure 11). The result remained essentially the same for models using a 2‐, 7‐, or 30‐day time interval to assess predator presence (Tables S1–S3).

**TABLE 1:** Model results for minimum distances between sleeping sites of parties in response to food availability and number of predator encounters within 2 weeks (estimates, standard errors, 95% credible intervals, Rhat, as well as bulk and tail effective sample sizes).

Term	Estimate	Est. error	CI_lower_	CI_upper_	Rhat	Bulk_ESS	Tail_ESS
Intercept	4.35	0.70	2.92	5.73	1.00	972	1653
pred.enc.14	−0.00	0.01	−0.03	0.03	1.00	1580	2138
food.score	0.29	0.87	−1.38	2.01	1.00	2424	2311

## 4 Discussion

### 4.1 Sleeping Sites and Habitat Types

Guinea baboons in Simenti spent most of their nights at sleeping sites in the gallery forest along the Gambia River. This frequent use of specific habitat types may reflect the availability of preferred sleeping tree species, such as *C. pentandra* , *C. integrifolia* , or *B. akeassii*, which predominantly occur in habitats close to perennial water bodies. Trees of these species might offer better protection against nocturnal predation than other tree species due to their height (15–20 m) and smooth bark or thorns around the trunk, making them more difficult for predators to climb (Sharman 1982; Anderson and McGrew 1984; Zinner et al. 2021). Sleeping trees located in the gallery forest with a canopy cover of 75%–100% (Klapproth 2010) may also provide better connectivity to surrounding trees, facilitating an escape during a nocturnal attack (Harrison et al. 2020). However, *B. akeassii* is abundant throughout the study area.

The intensive use of sleeping sites in gallery forests, wetlands, or temporary wetlands rather than in savannah habitats, where suitable sleeping trees such as *B. akeassii* are also abundant, may be associated with proximity to drinking water. During the dry season (November–May), the only available water sources in the study area are the Gambia River, one perennial wetland, and a few water bodies that usually dry out with the progression of the dry season. Sleeping near these water sources allows Guinea baboons to drink in the mornings and evenings. Similarly, Guinea baboons near Mont Assirik in the southeastern part of the Niokolo‐Koba National Park selected sleeping sites that were, on average, 5 m from water bodies (Sharman 1982).

This argument is weakened, however, by their increased use of sleeping sites in savannah woodlands during the dry season of 2022. In 2022, Guinea baboons spent, on average, 16.4% (mean, range 3%–29%) of their nights in sleeping sites located in savannah habitats (savannah woodland or tree/shrub savannah) compared to 0%–3% during the rainy season. This shift might be due to feeding trees and herbaceous food items being available in savannah habitats during these months, with tree species like *B. akeassii*, *Strychnos spinosa* , *Piliostigma thonningii* and *Acacia seyal* providing ripe fruit.

Our study parties may have used sleeping sites closer to these abundant food resources to minimise travel distances. This would reduce travel costs and allow more time and energy to be directed toward feeding, a pattern seen in other primates as well. Hamadryas baboons are thought to change their sleeping cliffs alongside changes in their foraging regions (Sigg and Stolba 1981) and occasionally sleep on doum palm trees (*Hyphaene* spp.). In this way, the baboons likely have easier access to highly valuable palm fruits when these are scarce elsewhere in their habitat (Schreier and Swedell 2008). As we lack finer‐scaled data on spatiotemporal food availability across our study parties' home ranges, this interpretation remains speculative. Further, the pattern we observed in 2022 was not evident in the other years of the study (2010–2012), suggesting that the variation in sleeping site use across habitats is likely driven more by opportunism than by successful resource exploitation and varies considerably between years, without any detectable consistent pattern.

### 4.2 Patterns of Sleeping Site Use of Individual Parties

Upon visual inspection of the distances between successive sleeping sites, it seemed as if Guinea baboon parties tended to use sleeping sites that were either very close to, or more than 3–4 km away from, previously used sites, with fewer sites used at intermediate distances. When comparing the observed patterns to the expected distribution assuming random visit orders, we found that the observed patterns did not coincide with any of the scenarios we initially proposed. None of our study parties showed a pattern of sleeping site use consistent with either the predator avoidance hypothesis or minimising parasite load from faecal matter build‐up. The only discernible trend was a slightly more frequent use of sleeping sites that were closer to previously used ones than would be expected by chance. This result suggests that predator presence or parasite load are not significant factors influencing the Guinea baboons' sleeping site use, at least not in the observed patterns. It is possible that other, less obvious factors are at play or that Guinea baboons in Simenti do not need to pay attention to these risks when selecting sleeping sites but instead use them opportunistically.

The parasite load hypothesis continues to be widely cited, often without direct testing, and is frequently assumed to play a relevant role even when results offer little support. Given the high prevalence of gastrointestinal parasites in Guinea baboons (up to 78%, N'da et al. 2022), the likely exposure to faecal matter across their range, and the high population density in the park (Galat‐Luong et al. 2006; Rabeil et al. 2018), it seems unlikely that switching sleeping sites meaningfully reduces infection risk. These factors suggest that the hypothesis may be overly simplistic and not universally applicable.

### 4.3 Distance to Other Parties

In 2022, Guinea baboon parties were almost always less than 50 m apart from another party, suggesting they may have even shared the same stand of trees. Further, most parties showed a strong preference to sleep closest to one or two specific other parties, except for P6I and P7, which exhibited more variable patterns. We did not, however, find any effect of food availability or predator presence on the distance between the sleeping sites of the closest parties. The lack of an observed effect of predator presence suggests that large aggregations at sleeping sites may not be related to communal predator detection or defence. It is essential to acknowledge that the absence of observed predators does not necessarily mean that predators were absent. Nocturnal predators such as leopards may have been nearby but undetected by the camera traps, observers, or the baboons themselves (Bidner et al. 2018).

Further, leopards preying on baboons at night do not seem to rely on surprise attacks (Busse 1980), suggesting that larger groups do not benefit substantially from enhanced detection or mobbing behaviour at night. However, these groups might still benefit from the dilution of risk, by which the probability of any individual being targeted by a predator decreases as group size increases (van Schaik 1983; Bidner et al. 2018). The formation of larger aggregations at sleeping sites (e.g., bands or gangs) may thus be primarily driven by the dilution of risk and social benefits. Larger aggregations at sleeping sites provide better opportunities for social interactions, offering more potential partners for socialising within and between social groups (Loftus et al. 2024). Guinea baboons often spend extended periods huddling, grooming and greeting each other, both within and between parties, at sleeping sites before starting their morning foraging activities (Sharman 1982; Patzelt et al. 2011). Interestingly, the observed tendency of certain parties to repeatedly sleep closest to particular others reflects patterns also seen during daytime associations, suggesting that the proximity between sleeping sites of parties is driven less by ecological factors and more by stable social preferences.

### 4.4 Limitations and Prospects

It is important to acknowledge several methodological shortcomings of this study. We were unable to identify individual sleeping sites or assess their specific properties, occupancies, and vacancies. Any attempt to delineate individual sleeping sites would have relied on arbitrary distance cut‐offs, given our study parties used nearly the entire extent of the gallery forests alongside the Gambia River as sleeping sites, particularly in the vicinity of the CRP Simenti campsite (Figure 2).

Therefore, our conclusions about sleeping site use are based on patterns of distances between successively used sleeping sites rather than on properties of individual sites or observed occupancy and vacancy patterns. Furthermore, we could not assess *sleeping site selection*, but only *sleeping site use*. That is, we do not know which sleeping sites or specific trees were available but not chosen, preventing us from evaluating selection criteria directly. Additionally, we were able to collar only a fraction of the baboon population present in the study area, suggesting that true patterns of sleeping site use may differ substantially from what we observed in our study parties. Sleeping sites might be used and reused at an even higher frequency than what we found in our study, which would offer even less support for the parasite avoidance hypothesis.

The lack of observed effects of food availability and predator presence in our models may partly result from the relatively coarse assessment of both, which may not have accurately captured changes in levels of competition or the perceived risk potentially affecting the sleeping site use of our study parties. Our assessment of food availability across the entire landscape likely did not appropriately capture the food‐related drivers of sleeping site selection, if there were any. While landscape‐level food availability may reflect competition between parties during their diurnal foraging activities in general, it is less suited to grasp the local distribution and abundance of resources potentially impacting sleeping site use. Future studies should incorporate direct observations of feeding behaviour and spatially explicit abundance and distribution of food resources at sleeping sites, both in the evenings before the baboons recede into a sleeping tree, as well as in the mornings as they leave.

Lastly, the patterns of sleeping site use we observed may not be solely determined by a given party's independent choices but are likely influenced by the movement decisions of other parties. Despite the wide availability of sleeping trees, parties consistently stayed close at night. Rather than being driven purely by the availability of sleeping sites, this cohesion may result from how parties navigate their daily travel routes, the timing of their arrival at sleeping sites, and whether other parties are already present. The consistent proximity between sleeping sites of parties highlights the need to further explore the social and spatial processes influencing sleeping site use. Future research should examine how space use and travel decisions during the day, particularly in the afternoons, shape sleeping site selection from a between‐party perspective.

## 5 Conclusion

Sleeping sites for Guinea baboons in Simenti are not a limited resource, and the baboon parties did not compete for them, as reported for other baboon species. Frequently used sleeping tree species, particularly *B. akeassii*, were available in most habitats and could be found across the entire study area in large numbers. We did not find any spatiotemporal patterns of sleeping site use that indicate predator avoidance or a reduction of parasite load at frequently used sites. We, therefore, conclude that the hypothesis of competition for sleeping sites and pronounced pressure from parasite infection is not supported by our findings for the Guinea baboons near Simenti. These baboons likely used the abundantly available sleeping sites opportunistically, rather than employing strategies aimed at predator avoidance or reducing parasite load. Future research should explore how social and spatial dynamics during the day shape sleeping site use from a between‐party perspective.

## Author Contributions

**Lisa Ohrndorf:** conceptualization (equal), data curation (equal), formal analysis (supporting), investigation (lead), visualization (equal), writing – original draft (lead), writing – review and editing (equal). **Roger Mundry:** data curation (equal), formal analysis (lead), visualization (equal), writing – original draft (supporting), writing – review and editing (equal). **Jörg Beckmann:** investigation (supporting), writing – original draft (supporting), writing – review and editing (equal). **Julia Fischer:** conceptualization (equal), funding acquisition (equal), supervision (supporting), writing – original draft (supporting), writing – review and editing (equal). **Dietmar Zinner:** conceptualization (equal), funding acquisition (equal), supervision (lead), writing – original draft (supporting), writing – review and editing (equal).

## Ethics Statement

The collaring of the baboons was approved by the Ethics committee of the German Primate Center (document number E4‐21), as well as by the Direction des Parcs Nationaux (DPN) and the Ministère de l'Environnement et de la Protéction de la Nature (MEPN) de la République du Sénégal (protocol d'accord from 22/04/2019).

## Conflicts of Interest

The authors declare no conflicts of interest.

## Supporting information


Data S1.

## Data Availability

All data and scripts for analyses are available on OSF (https://doi.org/10.17605/OSF.IO/2G9ER).
