# Supplementary material for: Spatiotemporal Patterns of Sleeping Site Use of Guinea Baboon Parties (Papio papio)
Source: Ecol Evol. 2025 Jul 27;15(7):e71610. doi: 10.1002/ece3.71610 (PMC12296831; doi:10.1002/ece3.71610)
Supplement: Supplementary file 1 — Data S1. [file ECE3-15-e71610-s001.pdf]

Supplementary material

Spatiotemporal patterns of sleeping site use of Guinea baboon parties (*Papio papio*)

Lisa Ohrndorf, Roger Mundry, Jörg Beckmann, Julia Fischer, Dietmar Zinner

Collaring procedure

Between January 16 and 21, 2022, we captured and collared eight Guinea baboons on five separate days. On each day, we located the target individuals and their parties in the morning and followed them until the target animal could be immobilised covertly (without the target or nearby group members seeing the shot). Immobilisation was achieved using a blowpipe (TeleDart B16, calibre 16 mm, length 160 cm) and 2 ml darts (BD2) with plastic stabilisers (BST16) and plain needles (1.5 × 38 mm, TDN1538LL). Each animal received an initial anaesthetic dose of 100 mg ketamine, 20 mg xylazine, and 2 mg atropine. Once fully sedated and with conspecifics at least 300 m away, we moved the animal to a shaded area, blindfolded it, and monitored vital signs (oxygen saturation, heart rate, and internal body temperature). We recorded body weight using a spring scale (average male body mass: 21.5 kg ± 1.9 SD; range: 19–25 kg; n = 8). After placing a collar, we positioned the animal in the shade near a small tree and administered 1.5 mg atipamezole as an antidote against xylazine. We then monitored the animals from a distance until they were fully awake and capable of moving safely. All individuals successfully rejoined their parties the same or following day. The entire procedure was accompanied by a veterinarian from the Senegalese Direction des Parcs Nationaux (DPN) and a representative of the Tambacounda veterinary service. For details on the collaring procedure from 2010 to 2012, see Knauf et al. (2015).

Table S1: Model results on minimum distances between sleeping sites of parties in response to food availability and number of predator encounters within 7 days (estimates, standard errors, credible intervals, Rhat, as well as Bulk and Tail Effective Sample Sizes).

Term	Estimate	Est.Error	CI_{lower}	CI_{upper}	Rhat	Bulk_ESS	Tail_ESS
Intercept	4.43	0.64	3.07	5.63	1.00	899	1760
pred.enc.7	-0.01	0.02	-0.05	0.02	1.00	2475	2792
food.score	0.37	0.82	-1.15	2.06	1.00	2605	2417

Table S2: Model results on minimum distances between sleeping sites of parties in response to food availability and number of predator encounters within 30 days (estimates, standard errors, credible intervals, Rhat, as well as Bulk and Tail Effective Sample Sizes).

Term	Estimate	Est.Error	CI_{lower}	CI_{upper}	Rhat	Bulk_ESS	Tail_ESS
Intercept	4.56	0.81	2.94	6.18	1.00	1065	1306
pred.enc.30	-0.01	0.01	-0.02	0.01	1.00	1667	1731
food.score	0.37	0.84	-1.25	2.11	1.00	2422	2315

Table S3: Model results on minimum distances between sleeping sites of parties in response to food availability and number of predator encounters within 2 days (estimates, standard errors, credible intervals, Rhat, as well as Bulk and Tail Effective Sample Sizes).

Term	Estimate	Est.Error	CI_{lower}	CI_{upper}	Rhat	Bulk_ESS	Tail_ESS
Intercept	4.30	0.55	3.18	5.40	1.01	1186	2043
pred.enc.2	0.01	0.04	-0.07	0.09	1.00	2268	2328
food.score	0.14	0.73	-1.25	1.65	1.00	3083	2714

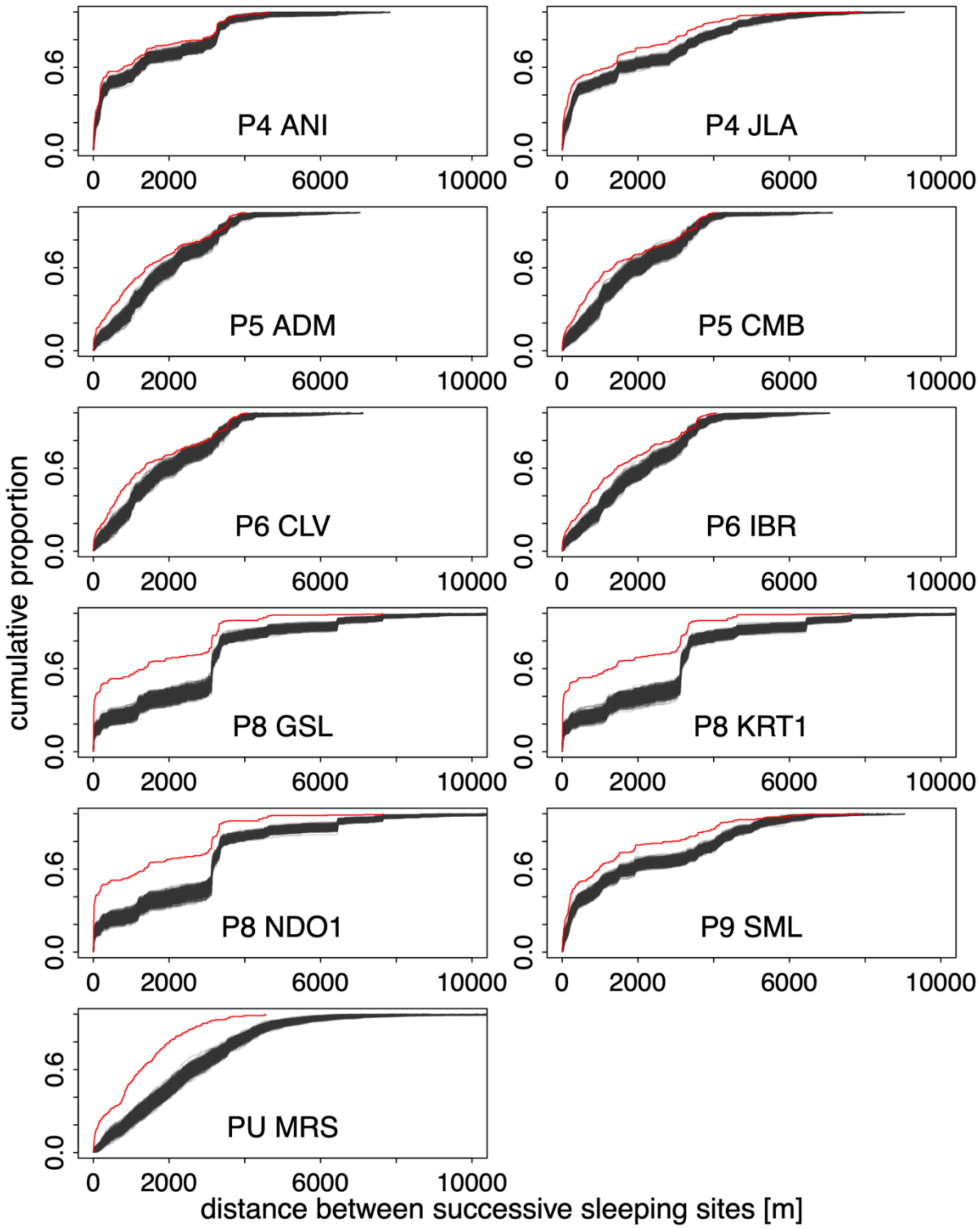

Figure S1: Patterns of sleeping site use of parties and associated collared individuals in 2010. Grey lines indicate 1000 randomised visit orders, and the red line depicts observed distances between successive sleeping sites.

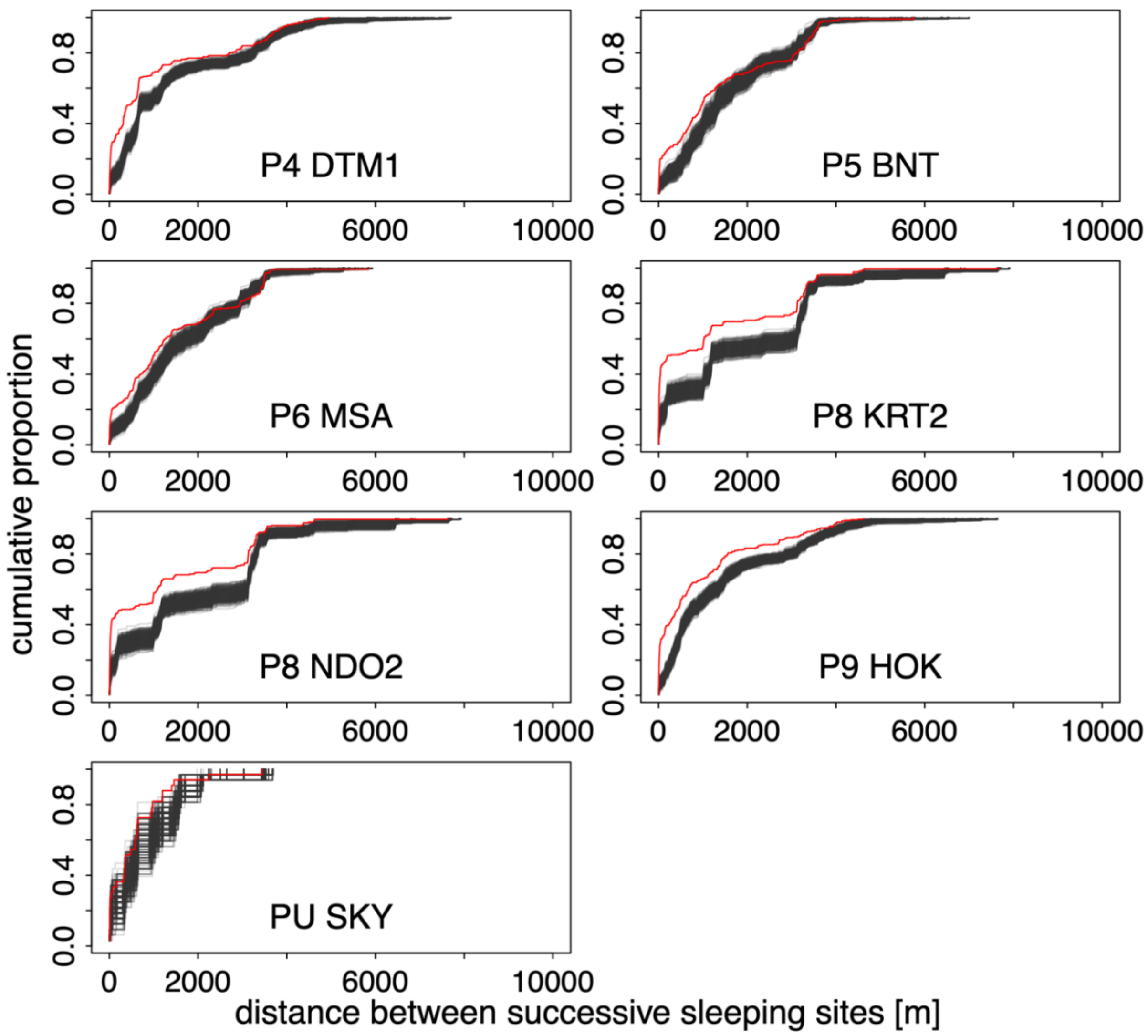

Figure S2: Patterns of sleeping site use of parties and associated collared individuals in 2011. Grey lines indicate 1000 randomised visit orders, and the red line depicts observed distances between successive night sleeping sites.

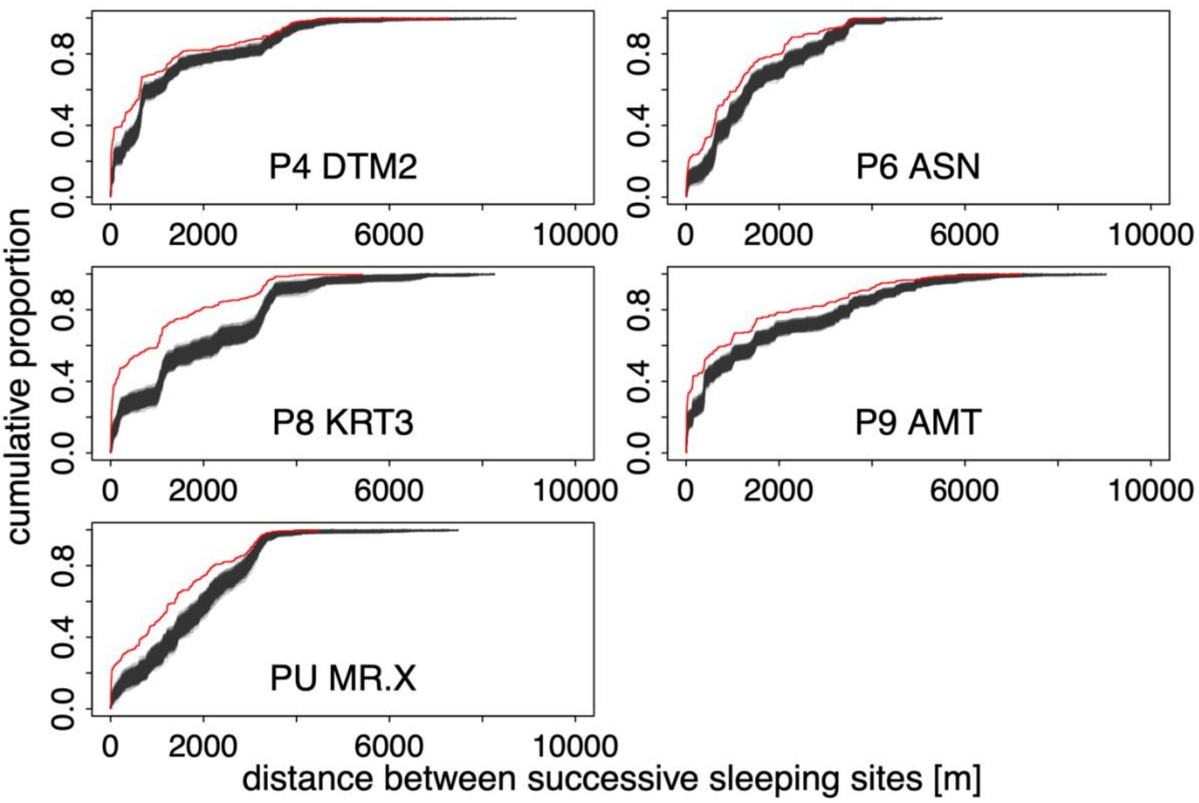

Figure S3: Patterns of sleeping site use of parties and associated collared individuals in 2012. Grey lines indicate 1000 randomised visit orders, and the red line depicts observed distances between successive sleeping sites.

References

Knauf, S., Barnett, U., Maciej, P., Klapproth, M., Ndao, I., Frischmann, S., Fischer, J., Zinner, D., & Liu, H. (2015). High prevalence of antibodies against the bacterium Treponema pallidum in Senegalese Guinea baboons (*Papio papio*). *PLOS ONE, 10*(11), e0143100. https://doi.org/10.1371/journal.pone.0143100
